# Supplementary material for: Content-rich biological network constructed by mining PubMed abstracts
Source: BMC Bioinformatics. 2004 Oct 8;5:147. doi: 10.1186/1471-2105-5-147 (PMC528731; doi:10.1186/1471-2105-5-147)
Supplement: Additional File 2 — The original results of the above study (non-essential files are deleted to keep the file size under the limit set by BMC bioinformatics). [file 1471-2105-5-147-S2.bz2 › chilibotAdditionalFile2/dip10/472ID9195914E10538/html/IL8RA_CXCL1.html]

 


 **IL8RA** and **CXCL1** 
  
Found 47 abstracts in PubMed, retrieved 10.  
 

 What does Google say? 
 PDF only 
| .edu only 

---

**Interactive relationship** (e.g. stimulation, inhibition, etc)

**Non-interactive relationship** (e.g. studied together, co-existance, homology, etc.)

- In addition, we demonstrate that compounds which inhibit the actions of IL 8 and GRO  [ **CXCL1** ]  alpha via their receptors, CXCR1  [ **IL8RA** ]  and CXCR2, also inhibit HIV 1 replication in both T lymphocytes and macrophages, indicating potential therapeutic uses for these compounds in HIV 1 infection and AIDS.  Ref: 11483765 J Virol, 2001
- Selective CXCR2 antagonists may be useful to block the Gro  [ **CXCL1** ]  alpha induced priming response whilst preserving neutrophil functions mediated by CXCR1  [ **IL8RA** ] , the alternative neutrophil receptor for interleukin 8.  Ref: 11589350 Eur Respir J, 2001
